# Heredity of Sebaceous Cysts

**Published:** 1882-12

**Authors:** 


### HEREDITY OF SEBACEOUS CYSTS.

In the course of a clinical lecture, reported in the *College and Clinical Record*, Prof. S. W. Gross says :

This man, who has a prominent swelling upon his scalp, tells me that his mother had a similar development. I asked him the question incidentally, because I had in mind the case of a man suffering with mammary cancer, upon whom I operated at this clinic, whose scalp was covered with these excrescences, and his daughter who came with him had also a number of them. Let me say in this connection that many surgeons mention as one of the points of difference between a malignant and a benign growth, that only the former is inherited, They seem to overlook that this also occurs in innocent tumors, even with the simplest of all, the sebaceous cyst.—*Med. and Surg. Rep.*

